# Kinetic Study on Chlorophyll and Antioxidant Activity from *Polyscias fruticosa* (L.) Harms Leaves via Microwave-Assisted Extraction

**DOI:** 10.3390/molecules26123761

**Published:** 2021-06-21

**Authors:** Thi-Thuy-Dung Nguyen, Quoc-Duy Nguyen, Thi-Van-Linh Nguyen

**Affiliations:** Faculty of Environmental and Food Engineering, Nguyen Tat Thanh University, Ho Chi Minh City 700000, Vietnam; dungntt@ntt.edu.vn (T.-T.-D.N.); nqduy@ntt.edu.vn (Q.-D.N.)

**Keywords:** chlorophyll, DPPH, microwave-assisted extraction, *Polyscias fruticosa* (L.) leaves, second-order kinetic model

## Abstract

*Polyscias fruticosa* (L.) leaves contain significant bioactive compounds with high antioxidant activity such as chlorophylls, total polyphenols, etc. but these have still been underutilized. In this study, the kinetics of chlorophyll and antioxidant activity extraction from *P. fruticosa* leaves by microwave-assisted extraction (MAE) were investigated. Microwave power was 300, 450, or 600 (W); the ratio of material/solvent varied from 1:40 to 1:80 (g/mL). In this study, the second-order kinetic model successfully predicted the change of chlorophyll and antioxidant activity during MAE. The increase of microwave power or/and the solvent amount increased saturated extraction efficiency and the extraction rate constant. However, the saturated concentration of chlorophyll and antioxidant activity increased with the increment of microwave power and the decrease in solvent amount.

## 1. Introduction

*Polyscias fruticosa* (L.) Harms belong to the *Araliaceae* family [1]. It is native to tropical Polynesian islands in the Pacific region [1] and is widely grown as an ornamental in Southeast Asian regions such as India, Malaysia, Indonesia [2], and Vietnam. Recently, *P. fruticosa* has been used as traditional medicine. Currently, this material still does not have a specific and clear chemical composition table. Scientists are still studying and synthesizing all the compounds in it. The roots and leaves of *P. fruticosa* contain saponins, alkaloids, several vitamins (vitamin B1, vitamin B2, vitamin B6, vitamin C), 20 kinds of amino acids, glycosides, cyanogenic glycosides, sterols, phytosterols, tannins, organic acids, essential oils, many trace elements and sugars (glucose, glucuronic acid, galactose, arabinose, rhamnose, etc.). The leaves contain triterpene saponins (about 1.65%), a genin that has been identified as oleanoic acid, but the nutrient content in leaves is less than that in roots [3,4,5]. About 24 compounds have been identified in the essential oil of its leaves. The main components are: β-elemene, α-bergamotene, germacren-d and (*E*)-γ-bisabolen [1]. In addition to saponins, flavonoids have also received the attention of researchers. Saito et al. (1990) [6] discovered two flavonoid compounds present in *P. fruticosa* leaves, namely kaempferol 3-*O*-α-L-rhamnopyranoside and quercitrin 3-*O*-α-L-rhamnopyranoside. In addition to determining the chemical composition of *P. fruticosa*, many studies have evaluated the pharmacological properties of extracts from other parts, including stems, roots, and leaves. Pharmacological effects have been detected from *P. fruticosa* such as anti-inflammatory and antibacterial abilities [7], improving asthma and respiratory symptoms [5], helping to limit sugar absorption [8], improving fertility [4,9], brain function [10], etc. Besides the applications of herbal medicine, *P. fruticosa* leaves are often used as salads in daily meals [11]. However, *P. fruticosa* leaves have been still underutilized.

Extraction is an important technique in the food industry. The extraction process could produce products or supply essential extracts that could improve the quality of the products. In green leaves such as *P. fruticosa* leaves, the natural green pigment is well-known as chlorophyll compounds that are extremely abundant worldwide [12]. In particular, chlorophyll has been proven to have high biological value with anticarcinogenic [13], anti-inflammatory, antibacterial [14,15], and antioxidant properties [13]. Furthermore, chlorophyll extracts could be used in a wide range of products, such as drinks, ice cream, sauces, pasta, and pickles. Therefore, extracts from *P. fruticosa* leaves containing chlorophyll, with antioxidant activity, could impart color or enhance the nutritional value in food products. While research on chlorophyll and antioxidant activity retention in final products is numerous, studies on the extraction of chlorophyll and antioxidant activity are still limited.

Many proposed extraction methods, especially techniques with the support of other processes, increase extraction efficiency, reduce time, or increase the ability to extract selectively. Among them, the microwave-assisted extraction (MAE) technique is considered to be a green technology [16] with numerous advantages such as reducing the use of organic solvents [16], shortening extraction time, and operating with lower temperature due to reducing the thermal gradients (so thermally labile compounds undergo less decomposition [17,18]). The solutes extracted by MAE are very varied. Although water is considered the compatible extraction solvent in MAE, MAE could also extract other compounds such as more medium- and non-polar organic compositions [19,20,21]. In the literature, many natural antioxidants have been successfully extracted with high speed and recovery by MAE, such as polyphenols and caffeine from green tea [22], ginsenosides from ginseng root [23], *E*- and *Z*-guggulsterone, cinnamaldehyde and tannin from the medicinal Asian plants [24], and flavonoids and phenolics from Chinese quince [25]. Extraction of chlorophyll and antioxidant activity from plants such as *Phyllanthus amarus*, *Adhatoda vasica*, *Tridax procumbens*, *Calotropis gigantea*, *Phyllanthus emblica L.*, *Gymnema sylvestre*, *Catharanthus roseus*, and *Psoralea corylifolio* were published in the previous study [26], but have still not been reported in *P. fruticosa* leaves.

Therefore, in this study, MAE of chlorophyll content and 2,2-diphenyl-1-picrylhydrazyl (DPPH) free radical scavenging activity from *P. fruticosa* leaves was investigated. Two important factors of the MAE technique, including microwave power (MW) and material/solvent ratio, were surveyed. Our work focused on identifying and evaluating kinetic parameters of MAE because these parameters play an essential role in the prediction of extraction process characteristics, allowing one to design experiments, optimize extraction conditions, and scale up the process.

## 2. Results and Discussion

### 2.1. Mathematical Models for MAE of P. fruticosa Leaves

**Figure 1:**
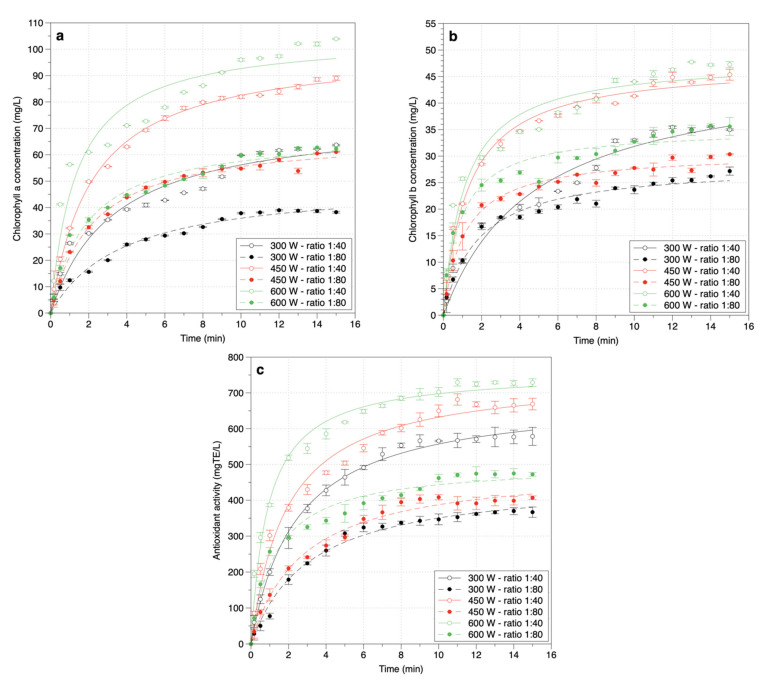
Changes in the concentration of chlorophyll a (**a**), chlorophyll b (**b**), antioxidant activity (**c**) during MAE of *P. fruticosa* leaves at different microwave power and material/solvent ratios.

The changes in chlorophyll a and b concentration and antioxidant activity during MAE of *P. fruticosa* leaves under different conditions are shown in Figure 1.

From the results of Figure 1, the chlorophyll (a and b) and antioxidant activity extraction curves in the study were consistent with the ideal 2-phase extraction curves of plant compounds. The initial phase of the process is the rapid extraction stage of chlorophyll and antioxidant activity, which quickly takes the first 6 min of extraction. In this stage, the solvents on the surface of the material are rapidly dissolved into the solvent. The second phase is the diffusion step, during which the rate of chlorophyll a and b concentration and antioxidant activity increases significantly compared to the first stage, and after a long time, the solid–liquid system will reach a saturation state [27]. MAE consumed a shorter time to complete the extraction process, which has been confirmed in many reports [28,29,30]. It is observed that MAE could extract astragalosides from *R. astragali* roots in a few min [28], coumarin, and o-coumaric acid from *Melilotus officinalis* (L.) *Pallas* in 10 min [29], or antioxidant from *Folium eriobotryae* in 3 min [30]. Compared to some previous studies on chlorophyll extraction via the conventional methods, MAE had significantly less extraction time. In this study, the MAE process could be stopped after 6 min, but the conventional methods needed from 1 to 4.3 h for extracting chlorophyll from rosemary leaves [31], and spinach by-products [32]. The very short operation time in MAE would bring more economic value. Therefore, MAE is a valuable extraction method for bioactive compounds.

**Figure 2:**
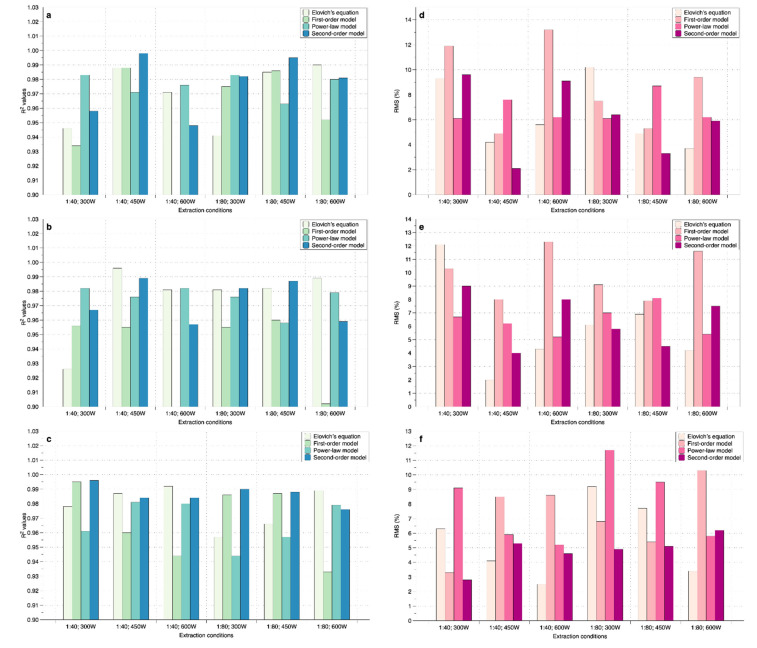
Comparison of model statistical parameters for MAE of *P. fruticosa* leaves: R^2^ value (chlorophyll a (**a**); chlorophyll b (**b**), antioxidant activity (**c**)); RMSE ((chlorophyll a (**d**); chlorophyll b (**e**), antioxidant activity (**f**)).

To analyze the behavior of chlorophyll and antioxidant activity extraction in MAE of *P. fruticosa* leaves, the tested models were evaluated for compatibility with experimental data. The model was compared based on statistics, including the lowest RMS and the highest R^2^ value, to choose which model was appropriate. The appropriate model will be selected to analyze the impact of the investigated factors on the desired responses. The results of the nonlinear regression analysis of mathematical models are presented in Figure 2.

Results from Figure 2 showed that R^2^ values were high (most were greater than 0.925), excepted the first-order model at 1:40 ratio, 600 W (chlorophyll a and b), 1:80 ratio, 600 W (chlorophyll b). For RMS, only the second-order model had an RMS of less than 10% for all extraction conditions, and the remaining models obtained some conditions with RMS greater than 10%. Specifically, the power-law model had an RMS of 11.7% for antioxidant activity extraction at 1:80; 300 W, the Elovich’s equation had RMS 10.2% and 12.1% for MAE of chlorophyll a (at 1:80; 300 W) and b (at 1:40; 300 W), respectively, and the first-order model had an RMS of more than 10% for both chlorophyll and antioxidant at 1:40 ratio (300 W and 600 W). The second-order model had R^2^ values higher than 0.948 for all extraction conditions. Therefore, in this case, the second-order model showed the best power of prediction. Many previous studies have confirmed that the second-order model had the best model in extraction processes such as convectional extraction [33,34,35,36], ultrasound-assisted extraction [37,38], high voltage electrical discharge extraction [39], and microwave-assisted extraction [40]. Therefore, in this study, the second-order model was used to characterize the behavior of chlorophyll and antioxidant activity extraction from *P. fruticosa* leaves via MAE.

### 2.2. Kinetics of Chlorophyll Content from P. fruticosa Leaves via MAE

From the second-order model, the parameters for chlorophyll extraction from *P. fruticosa* via MAE were estimated and are presented in Table 1.

Linear regression of the data from Table 1 (kinetics parameters of chlorophyll a and b extraction via MAE) could be represented in terms of material/solvent ratio and microwave power. Equations (1) and (2) show the correlation between the saturated concentration (C_s_, mg/mL), material/solvent ratio, and microwave power. The standard errors for the fit were approximately 7.18 and 1.69 mg/mL for chlorophyll a and b, respectively. The adjusted R^2^ values were 0.896 for chlorophyll a and 0.966 for chlorophyll b:

(1) $$C_{s}\left(\mathrm{chla}\right)\left(\mathrm{mg}/\mathrm{mL}\right)=77.62+15{.79x}_{1}+12{.36x}_{2}$$

(2) $$C_{s}\left(\mathrm{chlb}\right)\left(\mathrm{mg}/\mathrm{mL}\right)=39.51+8{.10x}_{1}+1{.92x}_{2}$$

where x_1_ and x_2_ are encoded levels of material/solvent ratio and microwave power, respectively.

Equations (3) and (4) gives a correlation of the extraction rate constant (k, mL/mg/min) for chlorophyll a and b, in which the adjusted R^2^ were 0.858 and 0.923, and the standard error was 5.534$\times 10^{-4}$ and 2.784$\times 10^{-3}$, respectively.

(3) $$k\;\left(\mathrm{chla}\right)\left(\mathrm{mL}/\mathrm{mg}/\min\right)=5.94\times 10^{-3}-6.867\times 10^{-4}x_{1}+1.327\times 10^{-3}x_{2}$$

(4) $$k\;\left(\mathrm{chlb}\right)\left(\mathrm{mL}/\mathrm{mg}/\min\right)=2\times 10^{-2}-7.027\times 10^{-3}x_{1}+6.815\times 10^{-3}x_{2}$$

Equations (5) and (6) give a correlation of the saturated extraction efficiency (Y_s_, mg/g) for chlorophyll a and b, in which the adjusted R^2^ values were 0.831 and 0.981, and the standard errors were 0.39 and 0.052, respectively.

(5) $$Y_{s}\left(\mathrm{chla}\right)\left(\mathrm{mg}/g\right)=4.34-0{.6x}_{1}+0{.68x}_{2}$$

(6) $$Y_{s}\left(\mathrm{chlb}\right)\left(\mathrm{mg}/g\right)=2.21-0{.3x}_{1}+0{.15x}_{2}-0{.12x}_{1}x_{2}$$

The coefficients in Equations (1)–(6) showed that, in MAE of *P. fruticosa* leaves, the increase in solvent amount decreased the saturated concentration (C_s_) but increased the saturated extraction efficiency (Y_s_) and the extraction rate constant (k) for chlorophyll a and b. The higher the microwave power was, the larger the values of saturated concentration (C_s_), saturated extraction efficiency (Y_s_), and the extraction rate constant (k) were. The highest values of Ys and k for chlorophyll concentration in MAE of *P. fruticosa* leaves were obtained at a microwave power of 600 W and a 1:80 ratio. In this study, when the solvent volume was kept constant for all runs, the changes of material/solvent ratio in experiments could be considered to absorb the insignificantly different energy from the same output microwave power. At low solvent ratios, it was reported that the diffusion of compounds from plant cells was controlled by mass transfer barriers [33,41]. Therefore, the extraction efficiency was low. When the solvent was used, a larger amount could increase the concentration gradient. Therefore, it would promote mass transfer [33], resulting in increased extraction efficiency. The extraction rate constant would also increase due to an increase in solute solubility at a higher amount of solvent. This was similar to the previous observation in which Mukhopadhyay et al. (2006) reported that when the ratio of material/solvent decreased, the extraction efficiency of phenolic compounds increased until the optimal value was reached [42]. For the saturated concentration of chlorophyll a and b, the results showed that when the ratio of material/solvent varied from 1:40 to 1:80 at the same microwave power, the values of chlorophyll saturated concentration decreased. The result was consistent with the extraction principle for solid–liquid systems [43]. However, the saturated extraction efficiency was larger at a 1:80 ratio. Therefore, if a high amount of solvent was used, the efficiency of the extraction process could be improved. Similar conclusions were confirmed by previous studies [40,44]. However, the effects of microwave power showed that the increase in microwave power made water molecules in the solid-liquid system absorb more radiation energy to transfer to thermal energy, leading to the temperature in the extraction system increasing very quickly. In addition, the interactions between solvent and solute could also increase the rate. Therefore, saturated concentration (C_s_), saturated extraction efficiency (Y_s_), and the extraction rate constant (k) had higher values at higher microwave power at a fixed ratio of material/solvent.

The MAE technique had a high extraction efficiency mainly due to microwave radiation with the volumetric heating through ion conduction and polar rotation mechanisms [45] causing a tremendous heating rate in MAE. Therefore, microwave power in MAE created the driving force of acceleration of molecular motion and internal diffusion more significantly than the conventional extraction process. Besides, the previous study reported that microwave power could disrupt plant cell structure [46], scorching, over-heating, charring, and even temperature distribution [47]; so the solute could be released easily. So, the high solubility concentration and extraction rate could be obtained in the MAE of *P. fruticosa* leaves. Some previous studies had similar conclusions that MAE had higher extraction efficiency than conventional extraction [48,49,50]. Pan et al. (2002) showed that MAE was the most efficient method for extracting tanshinones from the roots of *Salvia miltiorrhiza bunge* [48]. Because the MAE technique could complete extraction quickly and be less labor-intensive than conventional techniques [48]. Amarni and Kadi (2010) extracted oil from the olive cake, and their work showed that microwave-assisted solvent extraction provided better yields within very short times than conventional solvent extraction [49]. Dahmoun et al. (2015) obtained that extracted tannins and flavonoids using MAE were significantly higher than ultrasound-assisted extraction and conventional solvent extraction [50]. The higher extraction yield was supposed due to the mechanism of microwave heating [50,51].

### 2.3. Kinetics of Antioxidant Activity from P. fruticosa Leaves via MAE

From the second-order model, the parameters for antioxidant activity extraction from *P. fruticosa* via MAE was also estimated and is shown in Table 2.

Linear regression of the data from Table 2 (kinetics parameters of antioxidant activity extraction via MAE) correlate with the material/solvent ratio and microwave power. Equations (7)–(9) show the correlation between kinetics parameters, and material/solvent ratio, and microwave power.

(7) $$C_{s}\left(\mathrm{mg}/\mathrm{mL}\right)=609.91+125{.10x}_{1}+27{.83x}_{2}$$

(8) $$k\;\left(\mathrm{mL}/\mathrm{mg}/\min\right)=7.205\times 10^{-3}-3.583\times 10^{-5}x_{1}+4.18\times 10^{-4}x_{2}+3.465\times 10^{-4}x_{2}^{2}$$

(9) $$Y_{s}\left(\mathrm{mg}/g\right)=34.09-4{.69x}_{1}+1{.54x}_{2}$$

where x_1_ and x_2_ are encoded levels of material/solvent ratio, and microwave power, the adjusted R^2^ values and the standard errors for the fit were approximately 0.988 and 15.41 for saturated concentration (C_s_), 0.996 and 2.512$\times 10^{-5}$ for the extraction rate constant (k), and 0.982 and 0.71 for saturated extraction efficiency (Y_s_), respectively. Based on coefficients in the above Equations (7)–(9), it was found that when the material/solvent ratio changed from 1:40 to 1:80, the extraction rate constant and saturated efficiency increased significantly, but the saturated concentration decreased. When increasing the MW, the specific extraction parameters, including saturated concentration, saturated extraction efficiency, and extraction rate constant, increased. It was found that the trend effects of MW power, and the material/solvent ratio on antioxidant activity, were similar to the chlorophyll compound. In this study, the ability of free DPPH radical scavenging was used to determine the antioxidant activity in extracts. The higher the power of free DPPH radical scavenging was, the higher the antioxidant activity in the extracts was. Antioxidant activity in leaf extracts was mainly dependent on compounds in the extract. It was reported that bioactive compounds such as the polyphenolic family, vitamins (i.e., C, E, etc.), and phytopigments (carotenoids, chlorophylls, etc.) could act as antioxidants [52,53,54]. For green leaf herbs like *P. fruticosa* leaves containing significant chlorophyll content, the antioxidant activity may be directly related to chlorophyll compounds in the extracts. Chlorophyll a and b has been proven to show strong antioxidant activity compared to pheophytin a and pheophytin b [55]. From Table 3, it was confirmed that the ability of radical DPPH scavenging had a strong positive correlation with chlorophyll a and b in *P. fructicosa* leaves (*p* < 0.01). Therefore, in this study, MAE conditions applied on antioxidant activity were observed to be similar to chlorophyll a and b.

## 3. Materials and Methods

### 3.1. Material and Chemicals

Fresh *P. fruticosa* leaves were purchased from the company of medicine plants at Tay Ninh province, Vietnam. Only high-quality leaves with bright green color without visual defects were chosen. The leaves were then washed, drained, and sorted by size before drying. The leaves had a length of 4.2 ± 0.5 cm and an initial moisture content of 3.76 g/g dry basis (d.b.). To ensure the materials were uniform, the leaves were dried by hot air at 50 °C until the moisture content was approximately 0.053 g/g d.b. Then, the dried leaves were ground and passed through 16 mesh sieve to obtain powder used as materials. Moisture content was determined using the oven method (AOAC, 945.21).

Acetone, 2,2-diphenyl-1-picrylhydrazyl (DPPH), and 6-hydroxy-2,5,7,8 tetramethylchroman-2-carboxylic acid (Trolox) were purchased from Sigma-Aldrich (St Louis, MO, USA).

### 3.2. The Extraction Process

The MAE apparatus was built from the commercial microwave oven (model EMM2001W, Electrolux, Ho Chi Minh City, Vietnam). The magnetron could generate microwave power changing from 150 to 750 W. In this study, the *P. fruticosa* leaves were extracted with acetone 80%. The design of the experiment used was a full factorial design with two factors, including the MW power (300, 450, and 600 W) and the ratio of materials to solvent (1:40, and 1:80 g/mL). The ratio range was chosen because it ensures that the material is completely immersed in the extraction solvent and provides a uniform sample heating rate. The ratio of material/solvent can be changed by keeping the solvent volume unchanged (changing the sample weight) or keeping the volume constant (changing the solvent volume). In this study, the volume of the solvent was kept constant for all runs. The extraction process was observed during the first 15 min of extraction. The extract was analyzed for chlorophyll content and DPPH free radical scavenging activity.

### 3.3. Determination of Chlorophyll Content

The chlorophyll extract was measured at 663, 645 nm using a UV-Vis spectrophotometer (model UV-1800, Shimadzu Inc., Kyoto, Japan) and calculated according to a previous report by Kumar et al. [56]. The content of chlorophyll a and b was calculated according to the following equations:

(10) $$\mathrm{Chla}\left(\mathrm{mg}/L\right)=\left(-2{.59A}_{645}+12{.72A}_{663}\right)\mathrm{df}$$

(11) $$\mathrm{Chlb}\left(\mathrm{mg}/L\right)=\left(22{.9A}_{645}-4{.67A}_{663}\right)\mathrm{df}$$

A_645_ and A_663_ corresponded to absorbances measured at 645 and 663 nm, and df is the dilution factor.

### 3.4. Determination of Antioxidant Activity

The antioxidant activity of the extract was determined using DPPH free radical scavenging capacity [57]. The stock DPPH solution (24 mg/100 mL) was prepared in methanol and stored at 4 °C for 24 h. The reagent solution was further diluted with methanol to the absorbance of 1.10 ± 0.02 units at 515 nm using a spectrophotometer. The sample (150 μL) was added to 2850 μL of the DPPH solution and left to stand for 30 min in dark conditions. The absorbance was then taken at 515 nm using the spectrophotometer. The antioxidant activity of extracts was expressed as:

(12) $$\mathrm{Antioxidant}\;\mathrm{activity}\;\left(\mathrm{mgTE}/L\right)=\left(\frac{\left(\frac{A_{\mathrm{blank}}-A_{\mathrm{sample}}}{A_{\mathrm{blank}}}\right)-b}{a}\right)\mathrm{df}$$

A_blank_ and A_sample_ are the absorbances of the blank and sample, respectively, a and b are the slope and y-intercept that were determined from the equation with Trolox as the standard, and df is the dilution factor.

### 3.5. Mathematical Model for Extraction Process

Many mathematical theories have been established based on both theory and experiment to predict extraction processes. Many models have been applied and extensively studied for extraction curves. Kitanović et al. (2008) [58] and Chan et al. (2013) [59] have summarized the models that were succeeded in the kinetics study of conventional and assisted batch solvent extraction. In this study, the popular models in the literature review, including first-order, second-order, power-law models, and Elovich’s equation, were evaluated for their power of prediction in MAE of *P. fruticosa* leaf to choose the best fit model for further kinetic analysis.

#### 3.5.1. The First-Order Model

The first-order was expressed according to the work of Alara and Abdurahman (2019) [60]:

(13) $$C=C_{s}\left(1-\exp\left(-k_{1}t\right)\right)$$

where C is the chlorophyll content or antioxidant activity at different extraction times (mg/mL), C_s_ is the saturated concentration (mg/mL), and k_1_ is the first-order extraction rate constant (1/min).

#### 3.5.2. The Second-Order Model

The second-order model is well-known as the rate law or the hyperbolic model. This model was proven to adapt in the modeling of solvent extraction of active compounds from various plants. The second-order model can be used to investigate the degradation rate of active compounds in the solution [61]. The second-order model is shown as:

(14) $$C=\frac{C_{\infty }^{2}\mathrm{kt}}{1+C_{\infty }\mathrm{kt}}$$

where $C_{\infty }\;$is the saturated concentration (mg/mL), and k is the second-order extraction rate constant (mL/mg/min).

From the second-order kinetic model, the parameters for the extraction process for *P. fruticosa* leaves were determined as follows:

Saturated concentration (C_s_, mg/mL):

(15) $$C_{s}=C_{\infty }$$

Saturated extraction efficiency (Y_s_, mg/g):

(16) $$Y_{s}=\frac{C_{s}V}{m}$$

where V is the total extraction volume (mL), and m is the sample mass (g).

#### 3.5.3. The Power-Law Model

The power-law model is the empirical model with two parameters [62], which was used to reveal the transport mechanism of any active agent through non-swelling devices that were most suitable for *P. fruticosa* leaf. The power-law model is shown as:

(17) $$C={\mathrm{Bt}}^{n}$$

where B is a constant incorporating the characteristics of the carrier–active agent system, and n is the diffusional exponent [58].

#### 3.5.4. Elovich’s Equation

Elovich’s equation was developed when efforts to fit the data to first and second-order kinetic expressions produced poor fits in the study of Paterson et al. (1999) [63]. Elovich’s equation is presented below:

(18) $$C=E_{1}{+E}_{2}\mathrm{lnt}$$

where E_1_ and E_2_ are empirical constants.

Statistic parameters, including the determination coefficient (R^2^) and the root mean square (RMS), were used to identify the predictive ability of the tested models:

(19) $$R^{2}=1-\frac{\sum_{i=1}^{N}{\left(C_{\exp,i}-C_{\mathrm{pre},i}\right)}^{2}}{\sum_{i=1}^{N}{\left(\bar{C_{\exp}}-C_{\mathrm{pre},i}\right)}^{2}}$$

(20) $$\mathrm{RMS}\left(\%\right)=\sqrt{\frac{1}{N}\overset{N}{\underset{i=1}{\sum }}{\left(\frac{C_{\exp,i}-C_{\mathrm{pre},i}}{\bar{C_{\exp}}}\right)}^{2}}$$

### 3.6. Data Analysis

All experiments were conducted in triplicate. Values were calculated using Microsoft Excel (2016) and expressed as mean and standard deviation. MATLAB R2014 software was used to test empirical data with models based on the Levenberg–Marquardt method.

## 4. Conclusions

The kinetics study of chlorophyll content and the antioxidant activity from *P. fruticosa* leaves using the MAE technique was performed and evaluated. The experimental data in the extraction of chlorophyll and antioxidant activity from *P. fruticosa* leaves via MAE was best fit to the second-order model in comparison with the first-order, power-law model Elovich’s equation. Based on the second-order model, kinetics parameters were estimated. In this study, two investigated factors, including the MW power, and the material/solvent ratio, showed significant impacts on the MAE of *P. fruticosa* leaves. Responses including saturated concentration, saturated extraction efficiency, and the extraction rate constant were correlated to MW power and the material/solvent ratio using linear regression of data. The larger the microwave power, the higher the values of saturated concentration, saturated extraction efficiency, and the extraction rate constant for both chlorophyll and antioxidant activity. In addition, the increase of solvent amount (ranging from 1:40 to 1:80 g/mL) would increase the saturated extraction efficiency and extraction rate constant but decrease the saturated concentration. In this study, the extraction process with a large amount of solvent could improve the extraction efficiency of chlorophyll and antioxidant activity from *P. fruticosa* leaves using the MAE technique. However, it is necessary to consider some drawbacks in removing the solvent to collect products. This study provided critical empirical data in the MAE of alternative plant resources and the rule of effects of extraction conditions on kinetics parameters. This information could be applied to design and scale up the process by solving a multi-objective optimization problem with economic consideration.

## Figures and Tables

**Table 1:** Nonlinear regression analysis of mathematical models in both chlorophyll a and b extraction kinetics by MAE technique.

Material/Solvent Ratio (g/mL)	Microwave Power (W)	C_s_ (mg/mL)	K (mL/mg/min)	Y_s_ (mg/g)
1:40	300	74.71	$4.06\times 10^{-3}$	2.99
	450	100.44	$4.58\times 10^{-3}$	4.02
	600	105.06	$7.12\times 10^{-3}$	4.20
1:80	300	49.43	$5.39\times 10^{-3}$	3.95
	450	67.53	$6.85\times 10^{-3}$	5.40
	600	68.53	$7.64\times 10^{-3}$	5.48
1:40	300	47.05	$4.44\times 10^{-3}$	1.88
	450	47.53	$1.64\times 10^{-2}$	1.90
	600	48.25	$1.88\times 10^{-2}$	1.93
1:80	300	28.41	$1.97\times 10^{-2}$	2.27
	450	30.64	$2.95\times 10^{-2}$	2.45
	600	35.15	$3.26\times 10^{-2}$	2.81

**Table 2:** Nonlinear regression analysis of mathematical models in antioxidant activity extraction kinetics by MAE technique.

Material/Solvent Ratio (g/mL)	Microwave Power (W)	C_s_ (mg/mL)	K (mL/mg/min)	Y_s_ (mg/g)
1:40	300	692.23	$5.95\times 10^{-4}$	27.69
	450	751.78	$7.02\times 10^{-4}$	30.07
	600	761.03	$1.45\times 10^{-3}$	30.44
1:80	300	459.11	$7.03\times 10^{-4}$	36.73
	450	493.67	$7.39\times 10^{-4}$	39.49
	600	501.64	$1.52\times 10^{-3}$	40.13

**Table 3:** Correlation between chlorophyll compounds and antioxidant activity in MAE of *P. fruticosa* leaves.

Chlorophyll Compounds	Antioxidant Activity	
	r	*p*-Value (2-Tailed)
Chlorophyll a	0.976	0.000
Chlorophyll b	0.950	0.000

## Data Availability

Data is contained within the article.
